# FeMnO_3_: Synthesis, Morphology, Dielectric Properties, and Electrochemical Behavior Toward HER by LSV

**DOI:** 10.3390/nano16050310

**Published:** 2026-02-27

**Authors:** Mukhametkali Mataev, Zamira Sarsenbaeva, Marzhan Nurbekova, Ramachandran Krishnamoorthy, Bahadir Keskin, Moldir Abdraimova, Zhanar Tursyn, Karima Seitbekova, Zhadyra Durmenbayeva

**Affiliations:** 1 Department of Chemistry, Faculty of Natural Sciences, Kazakh National Women’s Teacher Training University, Almaty 050000, Kazakhstan; mataev.m@qyzpu.edu.kz (M.M.); abdyraimova.m@qyzpu.edu.kz (M.A.); janartursyn@gmail.com (Z.T.); seitbekova.k@qyzpu.edu.kz (K.S.); durmenbayeva.zh@qyzpu.edu.kz (Z.D.); 2 Department of Physics, SRM Institute of Science and Technology, Vadapalani Campus, Chennai 600026, Tamil Nadu, India; ramachak1@srmist.edu.in; 3 Department of Chemistry, Faculty of Arts & Science, Yildiz Technical University, Istanbul TR34220, Turkey; bahadirkeskin@gmail.com

**Keywords:** FeMnO_3_, perovskite-like, synthesis, dielectric properties, linear sweep voltammetry

## Abstract

This paper presents a comprehensive investigation into the synthesis, morphological characteristics, electrical conductivity, dielectric behavior, and electrocatalytic activity of perovskite-structured iron manganite (FeMnO_3_), with a specific focus on its performance in the hydrogen evolution reaction (HER). FeMnO_3_(FMO) nanoparticles (NPs) were synthesized using a sol–gel-type Pechini method and characterized by X-ray diffraction (XRD), Fourier-transform infrared spectroscopy (FT-IR), and field-emission scanning electron microscopy combined with energy-dispersive X-ray spectroscopy (FESEM-EDS). XRD analysis confirmed the formation of a crystalline structure with cubic symmetry assigned to the Ia-3 space group, with an average crystallite size of 52.47 nm. FESEM images revealed a relatively uniform morphology with an average particle diameter of 55.84 nm. The redox and oxidation states of Fe and Mn can be studied by temperature-programmed oxidation (TPO-O_2_) in order to understand oxygen uptake and metal oxidation processes occurring within the FMO lattice. The dielectric constant, dielectric loss, electric modulus and electrical conductivity were calculated as a function of frequency and temperature using a Novocontrol Alpha-A broadband dielectric spectrometer (Novocontrol system) coupled with the LCR-800 precision meter. The dielectric data reveal that the FMO has semiconducting behavior with dominant charge- or ionic-relaxation processes. The electrocatalytic activity toward the HER was evaluated using linear sweep voltammetry (LSV), with the working electrode modified by an FMO catalyst ink. The material exhibited significant catalytic activity within the HER potential range, and an increase in the number of cycles led to stabilized current and enhanced hydrogen evolution. These results highlight the stability of FeMnO_3_ for hydrogen generation.

## 1. Introduction

The general formula of a three-dimensional (3D) perovskite structure is ABO_3_, where A and B are metal cations, with the A-site cation typically larger than the B-site cation [1,2,3,4,5,6,7]. The A-site is usually occupied by a large-radius cation, such as an alkali, alkaline earth, or rare-earth metal. In perovskite structures, the B-site is typically occupied by transition metal oxides. Some transition metal oxides, such as manganese oxide, zinc oxide, and cobalt oxide, are widely used as anode materials in lithium-ion batteries due to their low cost, chemical stability, and relatively low activity compared to noble metals. These nanostructured materials are of great interest due to their unique physicochemical properties, which differ significantly from those of their bulk counterparts, largely owing to their high surface-to-volume ratio. This characteristic makes them suitable for various applications, including fuel cells, sensors, and catalysis. Particularly, bimetallic perovskite-type oxides such as FeMnO_3_ and SrMnO_3_ exhibit exceptional structural and functional properties [7,8]. The complex oxide mineral formed by manganese and iron oxides is known as bixbyite. FeMnO_3_ represents a rare example of bixbyite-type compounds composed of iron and manganese oxides. In nature, this rare mineral occurs at a Mn:Fe atomic ratio of approximately 75:25 [8,9]. Among various perovskite structures, FMO has attracted considerable research attention due to its natural abundance, low toxicity during synthesis, and excellent electrochemical properties [3,10]. In the FMO structure, Fe and Mn atoms are present in a 1:1 ratio and occupy the 8a and 24d Wyckoff positions, respectively, within a cubic Ia-3 crystal lattice [11,12,13].

At elevated temperatures, FMO demonstrates significantly enhanced dielectric conductivity, dielectric loss, and AC conductivity, which can be attributed to increased electron hopping mechanisms and interfacial polarization at the electrode–material interface. These features collectively indicate the semiconducting nature of the composition [8,14]. FMO exhibits complex dielectric behavior that is strongly dependent on frequency, temperature, humidity, and magnetic field. These characteristics make it a promising candidate for applications in energy storage and multifunctional devices where both dielectric and magnetic properties are crucial [15,16]. This compound exhibits ferrimagnetic behavior at room temperature and undergoes a transition to an antiferromagnetic state in the temperature range of 36–40 K. Dielectric studies have revealed phase transitions near 36 K and 100 K. The pronounced magnetic anisotropy and significant magnetocaloric effect observed in FMO suggest that it is a promising material for application in magnetic refrigeration technologies [6,9,11,17].

Hydrogen (H_2_) is considered a promising energy carrier due to its high specific energy density and is regarded as a key solution for reducing carbon emissions. This is because the use of hydrogen as a fuel does not produce carbon dioxide; instead, the only by-product is water vapor (H_2_O) [6,18]. Therefore, the advantages of hydrogen as a clean, versatile, and efficient energy source are most fully realized when it is produced from renewable energy sources. The hydrogen evolution reaction (HER) and the hydrogen oxidation reaction (HOR) are key electrochemical processes that play a crucial role in the hydrogen economy. The HER represents the cathodic half-reaction occurring during water electrolysis. At present, a major barrier to the widespread deployment of water electrolyzers and fuel cells is the sluggish kinetics of electrochemical reactions such as the HER and HOR. These slow reaction rates result in high overpotentials, leading to significant energy losses during the energy conversion process. To overcome these limitations, materials with high catalytic activity are required to enhance the efficiency of both the HER and HOR. Electrocatalysts offer a promising solution to this issue by accelerating electron transfer and reducing the activation energy of the reactions, making them a central focus in HER and HOR research. However, the most efficient catalysts for HER and HOR to date are noble metals such as platinum, which are scarce and expensive. The high cost and limited availability of these materials restrict their practical application in large-scale hydrogen technologies [19]. FMO has attracted considerable interest for catalytic hydrogen evolution due to its significant potential in the fields of catalysis and electrochemical engineering. The unique crystal structure of this perovskite-type manganite offers several advantages that enhance the efficiency of all stages of the electrocatalytic process [3,7]. FeMnO_3_-based nanostructures have been employed to modify fluorine-doped tin oxide (FTO) electrodes and have demonstrated high catalytic activity toward the oxygen evolution reaction (OER) in alkaline media [20]. The catalyst exhibits high stability and remarkable efficiency, making it a promising low-cost and alternative candidate for water electrolysis applications.

FMO nanocrystalline powder has also been investigated as an effective catalyst for the oxidation of ethanol, methanol, toluene, and xylene. Ethanol oxidation was observed at 230 °C, reaching a conversion rate of 97% at 300 °C. The catalyst demonstrated efficient performance at moderate temperatures, indicating its potential as a viable alternative to noble metal-based catalysts [21]. In Luria–Bertani (LB) medium, FMO nanoparticles inhibited the growth of Bacillus subtilis, even at low concentrations (0.01 mg/mL). This antibacterial effect is attributed to the release of toxic metal ions and the generation of reactive oxygen species (ROS). In contrast, no significant antibacterial activity was observed in Brain–Heart Infusion (BHI) medium, which is rich in proteins and oligosaccharides. These findings suggest that the antibacterial efficacy of FeMnO_3_ nanoparticles is highly dependent on the composition of the surrounding medium, which can influence their physicochemical properties [22]. The diverse functional properties of this compound highlight its potential as a versatile material for applications in multiple technological domains. This work is novel, as we prepared FMO nanomaterials and then carefully studied their structure, electrical properties, and how they promote reactions.

In this study, FeMnO_3_ was synthesized via the Pechini method with a calcination duration of 6 h. The work primarily focuses on a detailed investigation of the structural, electrical conductivity, and dielectric properties of the material. The electrochemical behavior of FeMnO_3_ under HER conditions is addressed at a preliminary level and assessed solely by LSV.

## 2. Materials and Methods

The following reagents were used: manganese(II) nitrate (Mn(NO_3_)_2_·xH_2_O, Buchs, Switzerland); iron(III) nitrate nonahydrate (Fe(NO_3_)_3_·9H_2_O, TU 6-09-02-553-96); citric acid (C_6_H_8_O_7_, GOST 908-79); and ethylene glycol (C_2_H_6_O_2_, GOST 10164-75).

Equipment and methods used: A laboratory-grade Brazilian agate mortar (diameter: 13 cm), and an SNOL laboratory muffle furnace were employed. Phase composition was determined using XRD analysis with a Miniflex 600 Rigaku (Tokyo, Japan), and morphological characterization was performed using FESEM, Thermo Scientific Apreo 2 S LoVac (Thermo Fisher Scientific, Hillsboro, OR, USA). FTIR spectra were recorded using a Bruker ALPHA FTIR spectrometer in the range of 400–4000 cm^−1^ (Ettlingen, Germany). TPO experiments were performed on a flow-type reactor attached to the not universal sorption gas analyzer USGA-101 (LLC “EkoKhim”, Moscow, Russia). The electrophysical properties of FeMnO_3_ were measured in the 293 K to 483 K temperature region and at frequencies of 1, 5, and 10 kHz using LCR-800 device (New Taipei City 236, Taiwan). Electrochemical measurements, including LSV, were carried out using a Reference 600 Potentiostat. Dielectric properties of FMO nanoparticles were comprehensively investigated using broadband dielectric and impedance spectroscopy. Measurements were conducted using Novocontrol concept 50 system (Montabaur, Germany) dielectric, conductivity, and impedance analyzer over the frequency range of 1 Hz to 3 MHz and temperature range of 293–373 K.

## 3. Results and Discussion

### 3.1. Synthesis of FeMnO_3_

**Figure 1:**
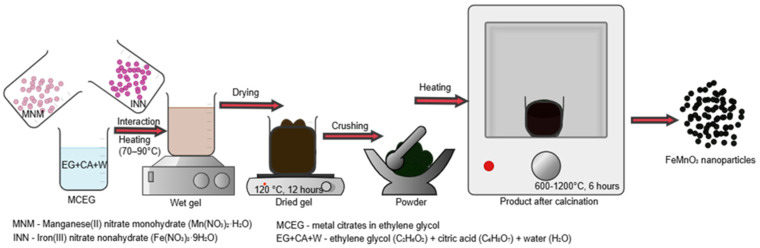
Step-by-step synthesis route of FMO nanomaterial via the Pechini-type sol-gel method [32].

The FMO nanomaterial was synthesized using the Pechini method based on the sol–gel process [23,24]. Many methods are known for the synthesis [25] of FMO nanomaterials: solid-phase precipitation [12], microwave synthesis [26], the hydrothermal method [20,27,28,29,30], the “green synthesis” method [1,3,25], urea combustion [31], methods based on sol–gel formation, etc. Among these methods, the reasons for choosing the Pechini (sol–gel-based) method are as follows: its formation of a homogeneous system with a uniform distribution characteristic of a complex oxide, the stoichiometrically accurate product, and the synthesis of nano-sized material. In this method, Mn^2+^ and Fe^3+^ ions form metal citrates with citric acid, which are reacted with ethylene glycol to form a uniform gel-like organic polymer. In this method, duration (6 h) and calcination temperature are used to obtain a truly crystalline nanoproduct from xerogel. Stoichiometric amounts of metal nitrates were used as precursors: 5.09 g of ferric nitrate nonahydrate (Fe(NO_3_)_3_·9H_2_O) and 2.47 g of manganese nitrate hydrate (Mn(NO_3_)_2_·xH_2_O) were weighed and dissolved in 10 mL of distilled water. To facilitate complexation and gel formation, 2.0 g of citric acid (acting as a chelating agent) and 2.72 mL of ethylene glycol (density: 1.1 g/mL) were added to the solution in a molar ratio of 1:1.5 with respect to the total metal cation content. The mixture was stirred magnetically at 70–90 °C for 10 min to initiate polyesterification and ensure homogeneity. The resulting sol was dried at 120 °C for 12 h to form a xerogel. The dried porous product was then ground into a fine powder and subjected to thermal treatment (calcination) at temperatures ranging from 600 to 1200 °C for 6 h in air (Figure S1). This process resulted in the formation of a single-phase perovskite-like FMO NP powder (Figure 1).

The elemental composition and crystalline structure of the synthesized material were characterized by XRD, FT-IR, and FESEM. Additionally, the dielectric and electrocatalytic properties of the material were systematically investigated.

### 3.2. XRD Analysis

XRD analysis was performed using a Rigaku MiniFlex 600 diffractometer (Tokyo, Japan). The measurements were conducted with CuKα radiation (λ = 1.5406 Å) over a 2θ range of 3° to 120°, with a step size of 0.01–0.02° and a scan time of 0.3–0.5 s per step. A nickel monochromator was used to filter the diffracted beam. The obtained diffraction data were analyzed using the PDF-5 database for phase identification. The average crystallite size of FeMnO_3_ was calculated using the Debye-Scherrer equation.

**Figure 2:**
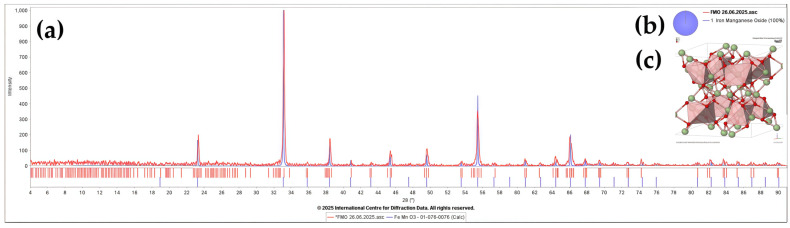
(**a**) XRD pattern of nanoproduct compared with reference (ICDD PDF 01-076-0076); (**b**) phase identification chart; (**c**) crystal structure model of FMO.

Table 1 presents the crystallographic parameters determined from the X-ray diffraction (XRD) analysis (Figure 2a), including the symmetry types of the identified phases (Figure 2b), lattice parameters, space group, calculated density values, and average crystallite sizes (D) (Figure 2c). Additionally, the diffraction peaks of the synthesized sample were analyzed using the PDF-5 database to confirm their correspondence to a perovskite-like structure. The high crystallinity of all samples is evidenced by the presence of sharp and well-defined diffraction peaks. The average crystallite size (D) was calculated using the Scherrer equation [33]:

D = k λ/β cos θ(1)

where k = 0.9400 is the shape factor, λ = 1.5406 Å is the wavelength of the X-ray radiation, β is the full width at half maximum (FWHM) of the most intense diffraction peak, and θ is the Bragg angle (in radians).

The average crystallite size corresponding to the diffraction peak of iron manganite was calculated to be 52.47 nm.

### 3.3. FT-IR Analysis

**Figure 3:**
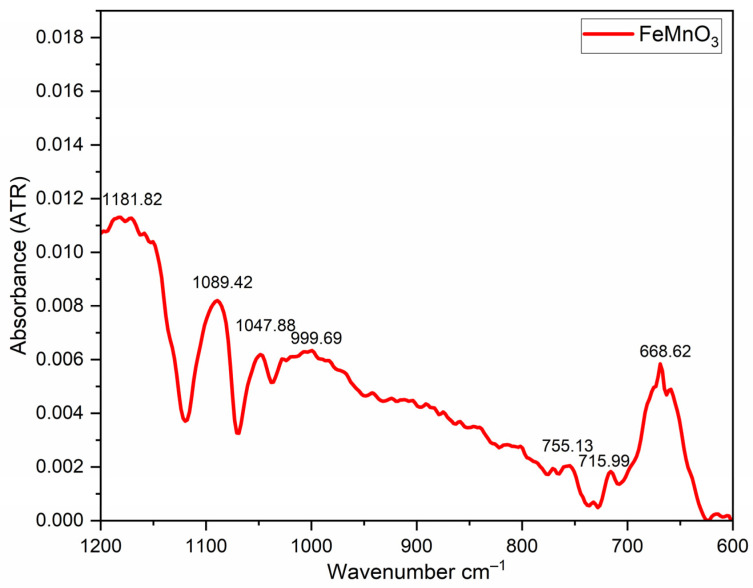
FTIR spectra of iron magnetite NPs.

FTIR spectroscopy was performed using a Bruker FTIR spectrometer equipped with an ATR module. The spectra were recorded in the range of 4000–400 cm^−1^ with a spectral resolution of 4 cm^−1^ and 32 scans per sample. The analysis was carried out at room temperature, and the data were processed using OPUS software. This technique allowed the identification of functional groups in the FMO material and monitoring of the thermal decomposition of residual organic compounds (Figure 3).

FTIR spectra characterize both the organic and inorganic components of the FeMnO_3_ compound synthesized via the Pechini method. The absorption bands in the range of 1181.82–999.69 cm^−1^ are associated with the vibrational modes of C–O, C–N, and C–C bonds, indicating the presence of residual organic precursors such as citric acid and ethylene glycol. In addition, the bands observed at 755.13, 715.99, and 668.62 cm^−1^ correspond to metal–oxygen (Fe–O and Mn–O) stretching vibrations, confirming the formation of the metal–oxide framework.

### 3.4. FE-SEM Analysis

The surface morphology of FMO fine powder was visualized and its elemental composition determined using field-emission scanning electron microscopy (FESEM). The analysis was performed with a Thermo Scientific Apreo 2 S LoVac instrument (USA). To evaluate the elemental distribution, the FESEM system was coupled with energy-dispersive X-ray spectroscopy (EDS). SEM images obtained at a scale of 500 nm revealed the surface structural features of the sample, while the EDS analysis provided quantitative information on the distribution of elements within the identified phases (Figure S2).

**Figure 4:**
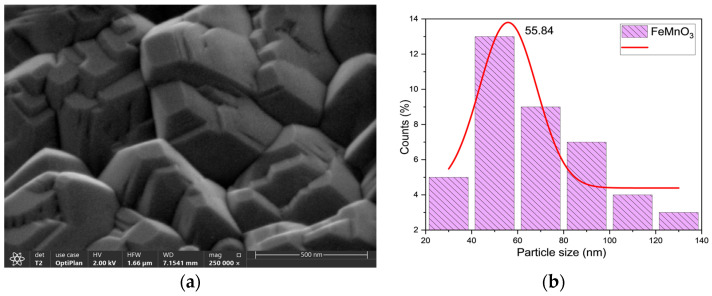
FESEM micrograph (**a**) and particle size distribution histograms (**b**) of FMO.

Figure 4 shows the FESEM image (a) and the particle size distribution (b) of the FMO sample. The surface dimensions of the polycrystalline particles were measured using ImageJ software. The elemental distribution and the average crystallite size were determined to be approximately 55.84 nm.

**Figure 5:**
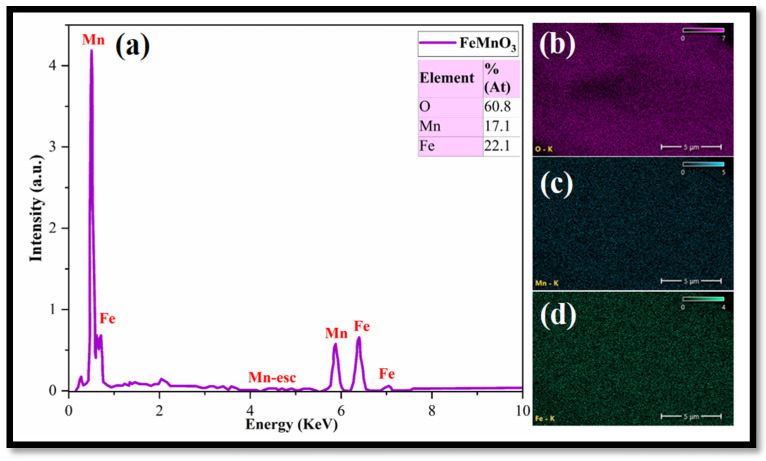
EDS (**a**) and elemental analysis mapping of FMO NPs: O (**b**), Mn (**c**), Fe (**d**).

Figure 5 shows the EDS spectra and elemental mapping of the synthesized FMO. The EDS spectra confirm the presence of key constituent elements (Fe, Mn, and O), verifying the qualitative and quantitative composition of the sample. The corresponding elemental mapping images demonstrate a homogeneous distribution of these elements across the surface, indicating uniform incorporation of metal cations within the matrix (Figure S3).

### 3.5. TPO-O_2_ Analysis of FeMnO_3_

Temperature-programmed oxidation (TPO) was conducted using a universal sorption gas analyzer (USGA-101, Moscow, Russia) equipped with a gas preparation system, a flow reactor with a tubular furnace (inner diameter 4 mm), and a thermal conductivity detector. Prior to the analysis, catalyst samples (63 mg, particle size 0.300–0.50 mm) underwent in situ pre-reduction in a hydrogen flow (30 mL min^−1^) at 465 °C for 40 min. After reduction, the samples were purged with helium and cooled to 50 °C at a cooling rate of 15 °C min^−1^. At 50 °C, the samples were held for 30 min, after which a He/O_2_ gas mixture (30 mL min^−1^, 5 vol.% O_2_ in He) was introduced, and the temperature was increased at a rate of 10 °C min^−1^ up to 950 °C. Upon completion of the TPO experiment, the samples were purged with helium and cooled to 100 °C at a rate of 15 °C min^−1^.

**Figure 6:**
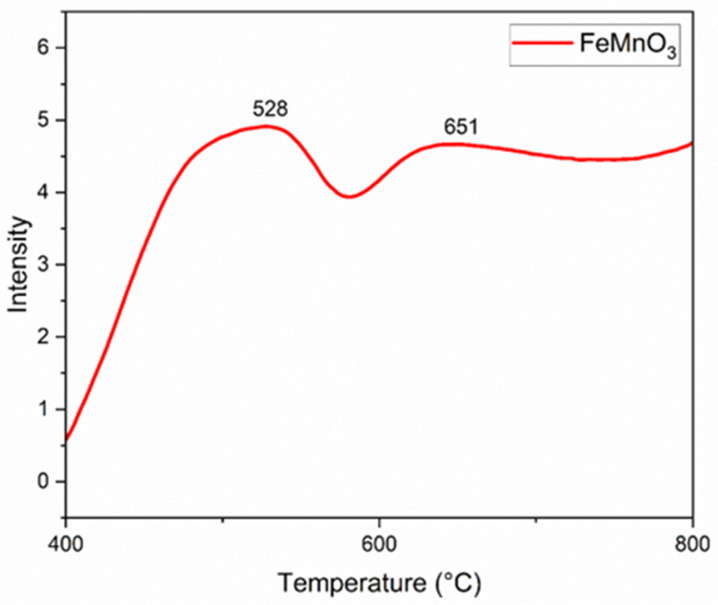
Temperature-programmed oxidation (TPO–O_2_) curve of FMO recorded in an O_2_ atmosphere.

The temperature-programmed oxidation (TPO) analysis for iron–manganese systems at specified temperatures describes the staged reduction in the material’s crystalline structure. At around 528 °C, some of the materialin the center oxidized. Then, between 400–550 °C, it absorbs oxygenas the metallic oxides (FeO) vanish, which results in simple oxides. At 651 °C, the more complex parts start to rebuild. The XRD data shows that the FMO structure starts appearing above 650 °C [34,35]. This indicates a complete regeneration of the material and restoration of its original crystalline lattice. The process is accompanied by a synergistic effect and electronic transition between manganese and iron ions (Fe^3+^ + Mn^2+/3+^ ↔ Fe^2+^ + Mn^4+^) [34,35,36] (Figure 6).

### 3.6. Preparation of a Tablet for Electrophysical Measurement

Electrophysical properties were measured using standard methods, focusing on the dielectric constant and electrical resistivity [37]. The electrical capacitance of samples was assessed with an LCR-800 instrument (Taiwan) at 1 kHz in continuous thermostat mode under dry air, maintaining fixed temperatures. Flat parallel disks (10 mm diameter, 2–6 mm thick) with approximately 1.5% binder were prepared, pressed at 20 kg/cm^2^, and fired at 400 °C for 6 h, followed by double-sided grinding. Dielectric permittivity was calculated from capacitance, using a Sawyer–Tower configuration to relate electrical induction D and electric field strength E. Observations were made with a C1-83 oscilloscope. Temperature was controlled using a chromel–alumel thermocouple, changing at approximately 5 K/min.

(2) $$\varepsilon =\frac{C}{C_{0}}$$

where $C_{0}=\frac{\varepsilon_{0}\cdot S}{d}$ is the capacitance of the capacitor without the investigated substance (air).

The calculation of the forbidden band width (ΔE) of the investigated substance was determined by the following formula [38]:

(3) $$\Delta E=\frac{2kT_{1}T_{2}}{0.43\times \left(T_{2}-T_{1}\right)}\mathrm{lg}\frac{R_{1}}{R_{2}},$$

where K is the Boltzmann constant equal to 8.6173303 × 10^−5^ eV·K^−1^ T, R_1_ is the resistance at T_1_, and R_2_ is the resistance at T_2_.

**Figure 7:**
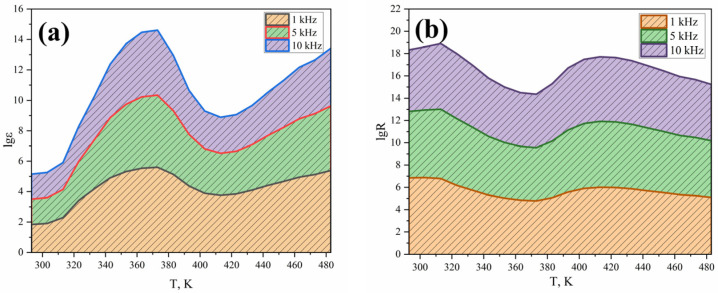
Dependence of dielectric permittivity (**a**) and electrical resistivity (**b**) of FMO on temperature at frequencies of 1, 5, and 10 kHz.

Electrophysical measurements of FMO in the range 293–483 K and frequencies equal to 1, 5, and 10 kHz were carried out on an LCR-800 setup (Figure 7).

The data of Figure 7 show that the value of ɛ equal to 69 at 293 K (1 kHz) reaches gigantic values up to 2.43 × 10^5^ when the temperature is increased to 483 K. When the frequency is increased to 10 kHz, the value of ɛ decreases, remaining relatively high at 483 K, equal to 6 × 10^3^.

The study of the temperature dependence of electrical resistivity on the temperature of material 2 shows a complex character of conductivity: in the interval 313–373 K—semiconducting, at 363–403 K—variable conductivity, and at 413–483 K—semiconducting (Table 2, Table 3 and Table 4).

FMO’s forbidden bandwidth is 0.12 eV between 313 and 373 K, 0.82 eV between 363 and 403 K, and 1.18 eV between 413 and 483 K. This is probably due to it being a narrow-bandgap semiconductor.

### 3.7. Dielectrical Measurements

Dielectric impedance measurements were carried out to investigate the dielectric and electrophysical properties of the samples using a Novocontrol Alpha-A broadband dielectric spectrometer. The measurements were performed over a frequency range of 1 Hz to 3 MHz and a temperature range of 293 K to 393 K (20 °C to 120 °C). The Quatro Cryosystem integrated into the device enabled precise control and stabilization of the sample temperature throughout the experiment. During each measurement cycle, the temperature was automatically adjusted in defined increments, and data acquisition was initiated only after the system stabilized at the target temperature.

Based on the obtained data, the real (ε′) and imaginary (ε″) parts of the dielectric constant, dielectric loss tangent (tan δ), electrical conductivity (σ), complex impedance (Z*), Nyquist plots, and relaxation-related parameters were calculated.

Dielectric conductivity is another important parameter associated with the dielectric relaxation process. Compared to dielectric loss values at 10 Hz, the dielectric conductivity is significantly lower (ranging from 10 to 1000), indicating that it decreases gradually with increasing frequency and drops to around one in the frequency range above 100 Hz. At lower frequencies, grain boundaries exhibit higher reactivity. At higher frequencies, a different behavior emerges, and the dielectric conductivity at f = 1 MHz becomes nearly temperature-independent.

**Figure 8:**
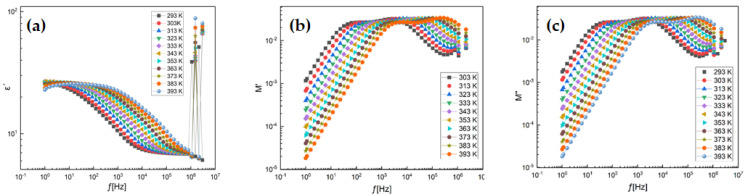
(**a**) Real part (ε′) of dielectric permittivity, (**b**) electric modulus behavior and (**c**) variation in imaginary part of modulus of FMO.

The temperature-dependent dielectric loss (tan δ) tends to decrease gradually with increasing temperature, except at lower frequencies (e.g., 6300 Hz) and temperatures up to 250 °C. In dielectric materials, the presence of defects, formation of space charges, and lattice distortion at grain boundaries can induce absorption currents, leading to dielectric losses. In NPs, the primary source of dielectric losses is associated with photon absorption due to structural imperfections, such as oxygen vacancies induced by doping (Figure 8a).

In Figure 8b, when data are represented using the modulus formalism, the influence of conductivity can be significantly suppressed. The modulus-based approach originated from the concept of considering the reciprocal of complex conductivity as the electrical analog of the mechanical shear modulus. Physically, the electric modulus corresponds to the relaxation of the electric field within the material under a condition of constant electric displacement. Thus, the modulus reflects the intrinsic relaxation process of the dielectric. To characterize the dielectric response of insulating materials, the complex electric modulus M’ was introduced. This formalism was later extended to materials with non-zero conductivity. The usefulness of the modulus representation in analyzing relaxation properties has been demonstrated for both glassy ionic conductors and NPs.

Figure 8c illustrates the typical frequency dependence of the imaginary part of the elastic modulus (M″). The imaginary component of the modulus increases consistently with frequency. Two plateau regions are observed between two distinct relaxation dynamics, which appear as two peaks in the M″ spectra. The low-frequency peak is associated with long-range charge hopping of carriers between adjacent ionic regions, corresponding to transitions at the boundaries between Mn^2+^/Mn^4+^ and Fe^2+^/Fe^3+^ sites. In contrast, the high-frequency peak is attributed to short-range hopping of charge carriers between spatially confined ions, enabling localized motion within the particles. The shift in the peak positions toward higher frequencies with increasing temperature indicates that at elevated temperatures, short-range hopping becomes more dominant.

**Figure 9:**
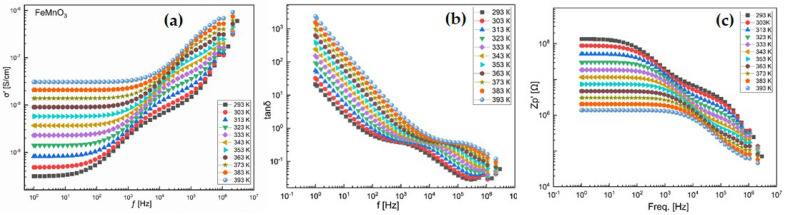
Frequency dependence of dielectric permittivity (**a**) and electric modulus (**b**,**c**) of FMO in the temperature range 293–393 K.

Figure 9a presents the frequency (f) dependence of the real part of the conductivity at selected temperatures in the range of 293 K to 393 K. The samples exhibit frequency-independent conductivity in the low-frequency region. The highly insulating nature (with low conductivity in the range of 10^−2^ to 10^−6^ S/cm) and the stable conductivity over a wide low-frequency range make these materials promising candidates for the design of high-quality resistive components.

The dielectric loss (tan δ) indicates a rapid decrease in dielectric losses with increasing frequency, dropping from values above one to below one around 100 Hz. At lower frequencies, the response is primarily governed by the grain boundaries of the highly disordered particles. The heterogeneous electronic structure (due to the d-electrons of transition metals) at the grain boundaries hinders the orientation of interfacial dipoles or the hopping process of charge carriers. This leads to greater absorption of electrical energy as current flows through the material (Figure 9b).

**Figure 10:**
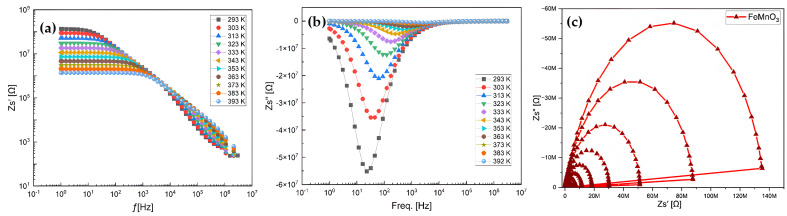
Variation in real (**a**) and imaginary (**b**) parts of the surface impedance with frequency and Nyquist plot (Zs″, Zs′) (**c**) at 293–393 K temperatures for FMO NPs.

To gain insight into the dynamics of mobile ions in the NPs, the real part of the complex electric impedance (Zp′) of the aforementioned NPs was plotted as a function of angular frequency at various temperatures, as shown in Figure 10. The plots exhibit typical curve behavior. Temperature has a significant effect on the resistance magnitude. At lower temperatures, Zp′ decreases monotonically with increasing frequency up to a certain point, after which it becomes frequency independent. High Zp′ values at low frequencies and low temperatures indicate strong polarization. The temperature at which this change occurs varies with frequency. This also suggests that the resistive grain boundaries become conductive at these temperatures. Furthermore, it indicates that the grain boundaries do not relax even at very high frequencies, even under elevated temperatures (Figure 9c).

To gain insight into the dynamics of mobile ions in FMO NPs, the imaginary part (Z″) of the complex impedance was plotted as a function of angular frequency at various temperatures, as presented in Figure 10a,b. The plots exhibit typical frequency-dependent behavior. Temperature has a pronounced effect on the magnitude of the impedance. At lower temperatures, Z″ decreases monotonically with increasing frequency up to a certain threshold, beyond which it becomes nearly independent of frequency. The high Z″ values observed at low frequencies and low temperatures indicate significant polarization within the material. The transition temperature at which this behavior changes is frequency-dependent, implying that the resistive grain boundaries become conductive at elevated temperatures. Additionally, the results suggest that grain boundaries do not undergo relaxation, even at very high frequencies and high temperatures.

The types of chemical bonds present play a crucial role in determining the electrical properties of NPs and their components. In a wide range of NPs that exhibit ionic conductivity, mixed ionic–electronic conductivity, or purely electronic conductivity, grain boundaries act as barriers to the transverse motion of charge carriers. The blocking nature of these grain boundaries is particularly pronounced at low temperatures. In this regime, perovskite-type manganites such as FMO are used as high-permittivity dielectrics with considerable conductivity for capacitor applications. At elevated temperatures (i.e., well above the Curie temperature), FMO-based NPs exhibit grain boundary (GB) layers with high resistivity, which is typically explained by the space charge depletion effect of the Schottky type. For most of the aforementioned applications, manganites must be purely acceptor-doped to prevent semiconducting-type conduction in the NPs’ bodie (Figure 10c).

### 3.8. LSV Analysis

**Figure 11:**
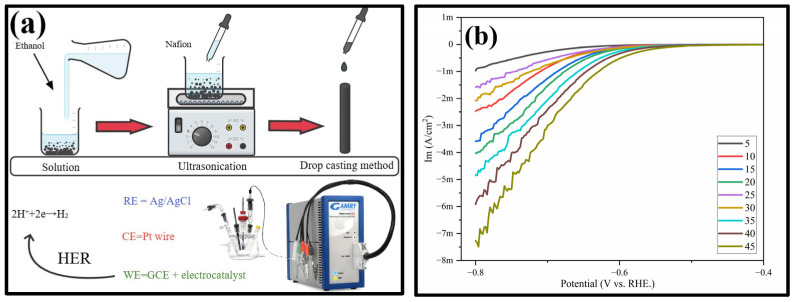
Preparation of working electrodes using the drop-casting method (**a**) and LSV curves (**b**) of the FMO composite.

The electrocatalytic properties of the synthesized materials were evaluated using a standard three-electrode system, consisting of a reference electrode (Ag/AgCl), a counter electrode (platinum plate), and a working electrode (glassy carbon electrode, GCE; surface area: 0.0314 cm^2^). Measurements were performed in 0.5 M H_2_SO_4_ solution using a Gamry electrochemical workstation (Reference 600 Potentiostat) to assess the bifunctional electrocatalytic activity. Prior to use as the working electrode, the GCE was polished with 0.05 µm alumina powder and then cleaned in an ultrasonic bath for 5 min in a mixture of ethanol and distilled water (EtOH:H_2_O = 1:3). To prepare the catalytic ink, 5 mg of FMO composite and 2 mg of carbon black were dispersed in 1 mL of deionized water and sonicated for 30 min to obtain a homogeneous suspension. Subsequently, 15 µL of the resulting catalytic ink and 10 µL of Nafion-117 solution were drop-cast onto the pretreated GCE surface and allowed to dry naturally at room temperature. Linear sweep voltammetry (LSV) measurements were then carried out in 0.5 M H_2_SO_4_ solution within the appropriate potential range to investigate the hydrogen evolution reaction (HER) activity (Figure 11 and Figure S3) [23].

LSV curves were recorded in the potential range of –0.8 to 0.4 V at a scan rate of 5 mV/s. From the voltammograms shown below, an increase in current within the –0.8 to 0.4 V range indicates hydrogen evolution occurring at the electrode surface. In the initial cycles (cycles 5 to 30), the curves display irregularities; however, with increasing cycle number, the current response becomes more stabilized. The lack of stability in the current during the early cycles may be attributed to side reactions occurring on the electrode surface. One of the primary causes of HER current instability is the formation of gas bubbles at the electrode surface, which leads to fluctuations in concentration overpotential and affects current distribution [39,40,41]. This observation suggests enhanced hydrogen evolution at higher cycle numbers. Additionally, current instabilities observed during the initial cycles may be influenced by over-limit diffusion currents due to electroconvection, as well as water dissociation-related effects [42,43,44], and parasitic electrochemical reactions associated with oxygen evolution and phase transitions [45,46].

The improved HER current density in a continuous mode of cycling could result from both the inherent activation of the catalytic layer and, when using a Pt counter electrode, Pt deposition. From the literature, it was previously reported that if Pt dissolution occurs followed by its redeposition on the WE (Pt ad-atoms), then an apparent increase in HER currents may be observed, and, for this reason, control measurements using non-platinum CEs are normally suggested to be performed in order to check that this effect is excluded [47]. On the other hand, we also note that increased HER currents can reflect real catalytic activation due to advance active site exposure and/or exalted reaction kinetics. In FMO-containing systems, lower charge transfer resistance and improved HER kinetics were also known [7], suggesting the potential for intrinsic catalytic activation. In the current research, the HER was noticed only after cast deposition of the FMO-coated catalytic layer on GC electrode, confirming its direct involvement in catalysis.

## 4. Conclusions

In this study, FMO perovskite-like NPs were synthesized via a sol–gel method based on the Pechini process, and their structural, morphological, dielectric, and electrocatalytic properties were systematically investigated. XRD analysis confirmed the formation of a cubic structure with space group Ia-3 and an average crystallite size of 52.47 nm. FTIR spectroscopy confirmed the complete decomposition of organic precursors and the formation of Fe–O and Mn–O bonds. FESEM revealed an average particle size of approximately 55.84 nm. We used TPO-O_2_ to figure out how manganese and iron change oxidation states during the reaction: Fe^3+^ + Mn^2+/3+^ ↔ Fe^2+^ + Mn^4+^. This enabled us to get a better grip on their electronic states and oxidation. The results demonstrate the preparation of phase-pure perovskite FMO with reliable structural and redox explanations.

Electroconductivity analysis showed that FeMnO_3_ has forbidden bandgaps of 0.12 eV (313–373 K), 0.82 eV (363–403 K), and 1.18 eV (413–483 K). This suggests that it is a narrow-bandgap semiconductor, which could explain how it acts. Dielectric spectroscopy results showed that the dielectric constant, dielectric loss, and AC conductivity of the iron manganite material were strongly dependent on both temperature and frequency. Impedance and electric modulus analyses provided insights into ion relaxation and intergranular polarization mechanisms. Nyquist plots and complex impedance values indicated that the material exhibits semiconducting behavior. Overall, these results suggest that FMO shows temperature- and frequency-dependent electrical and dielectric behavior dominated by semiconducting transport and an intergranular polarization mechanism.

LSV was employed to assess the performance of the nanomaterial in the HER. Upon application of the perovskite-based FMO catalyst to the electrode, a substantial current was recorded in the HER area. The current got steady after a few cycles, which tells us that the catalyst is stable. FMO NPs act like a semiconductor and show moderate performance as a catalyst for HERs. In summary, the LSV results demonstrate that FMO NPs possesses stable electrochemical performance upon initial activation with semiconducting behavior and moderate catalytic efficiency in HERs.

## Figures and Tables

**Table 1:** Crystallographic parameters obtained from X-ray diffraction analysis.

№	Name of Compound	Types of Syngony	a, Å	b, Å	c, Å	Vc., (Å^3^)	Z	D (Crystal Size, nm)	Space Group	Density (ρx-Ray, g/cm^3^)
1	FeMnO_3_	cubic	9.45	9.45	9.45	846.18	16	52.47	Ia-3 (206)	4.98

**Table 2:** Calculation of the forbidden band width in the interval 313–373 K.

T, K	lg R
313	6.79
373	4.78
$\Delta E=\frac{2\times 0.000086173\times 313\times 373}{0.43\times (373-313)}\mathrm{lg}\frac{6.79}{4.78}=0.12\;\mathrm{eV}$	

**Table 3:** Calculation of the forbidden band width in the interval 363–403 K.

T, K	lg R
363	4.85
403	5.90
$\Delta E=\frac{2\times 0.000086173\times 363\times 403}{0.43\times (403-363)}\mathrm{lg}\frac{4.85}{5.90}=0.82\;\mathrm{eV}$	

**Table 4:** Calculation of the forbidden band width in the interval 413–483 K.

T, K	lg R
413	6.01
483	5.10
$\Delta E=\frac{2\times 0.000086173\times 413\times 483}{0.43\times (483-413)}\mathrm{lg}\frac{6.01}{5.10}=1.18\;\mathrm{eV}$	

## Data Availability

The datasets used and analyzed during the present study are available from the corresponding author on reasonable request.

## Supplementary Materials

The following supporting information can be downloaded at: https://www.mdpi.com/article/10.3390/nano16050310/s1, Figure S1: Images of FeMnO_3_ nanopowder calcined at 120, 600, and 1200 °C; Figure S2: EDS point analysis results (a) and FESEM images (b) of FMO; Figure S3: Mapping analysis results of FMO (a). Histogram of the particle size distribution of the samples determined from FESEM micrographs of FMO (b); Figure S4: Photo images of the GCE modified with FMO NP material; Table S1: Electrophysical measurement results of FMO obtained in the temperature range of 293–483 K and at frequencies of 1(a), 5(b), and 10(c) kHz.
