# Supplementary material for: FeMnO3: Synthesis, Morphology, Dielectric Properties, and Electrochemical Behavior Toward HER by LSV
Source: Nanomaterials (Basel). 2026 Feb 27;16(5):310. doi: 10.3390/nano16050310 (PMC12986709; doi:10.3390/nano16050310)
Supplement: Supplementary file 1 [file nanomaterials-16-00310-s001.zip › nanomaterials-4148819-supplementary.pdf]

Supporting Information

$FeMnO_3$: Synthesis, Morphology, Dielectric Properties, and Electrochemical Behavior Toward HER by LSV

Mukhametkali Mataev [1], Zamira Sarsenbaeva [1,*], Marzhan Nurbekova [1,*], Ramachandran Krishnamoorthy [2], Bahadir Keskin [3], Moldir Abdraimova [1], Zhanar Tursyn [1], Karima Seitbekova [1] and Zhadyra Durmenbayeva [1]

[1] Department of Chemistry, Faculty of Natural Sciences, Kazakh National Women's Teacher Training University, Almaty 050000, Kazakhstan; mataev.m@qyzpu.edu.kz (M.M.); abdyraimova.m@qyzpu.edu.kz (M.A.); janartursyn@gmail.com (Z.T.); seitbekova.k@qyzpu.edu.kz (K.S.); durmenbayeva.zh@qyzpu.edu.kz (Z.D.)

[2] SRM Institute of Science and Technology, Vadapalani Campus, Chennai 600026, Tamil Nadu, India; ramachak1@srmist.edu.in

[3] Department of Chemistry, Faculty of Arts & Science, Yildiz Technical University, Istanbul TR34220, Turkey; bahadirkeskin@gmail.com

* Correspondence: sarsenbayeva.z@qyzpu.edu.kz (Z.S.); nurbekova.m@qyzpu.edu.kz (M.N.)

Experimental sections

Materials: The following reagents were used: manganese(II) nitrate ($Mn(NO_3)_2 \cdot xH_2O$, Buchs, Switzerland); iron(III) nitrate nonahydrate ($Fe(NO_3)_3 \cdot 9H_2O$, TU 6-09-02-553-96); citric acid ($C_6H_8O_7$, GOST 908-79); and ethylene glycol ($C_2H_6O_2$, GOST 10164-75).

Equipment and methods used: A laboratory-grade Brazilian agate mortar (diameter: 13 cm), and a SNOL laboratory muffle furnace were employed. Phase composition was determined using XRD analysis Miniflex 600 Rigaku (Tokyo, Japan), and morphological characterization was performed using FESEM, Thermo Scientific Apreo 2 S LoVac (USA). FTIR spectra were recorded using a Bruker ALPHA FTIR spectrometer in the range of 400–4000 cm^{-1} (Ettlingen, Germany). TPO experiments were performed on a flow-type reactor attached to the not universal sorption gas analyzer USGA-101 (Moscow, Russia). The electrophysical properties of $FeMnO_3$ were measured in the 293 K T 483 K temperature region and at a frequency of 1, 5, and 10 kHz used LCR-800 device (Taiwan). Electrochemical measurements, including LSV, were carried out using a Reference 600 Potentiostat. Dielectric properties of FMO nanoparticles were comprehensively investigated using broadband dielectric and impedance spectroscopy. Measurements were conducted using Novocontrol concept 50 system (Germany) dielectric, conductivity, and impedance analyzer over the frequency range of 1 Hz to 3 MHz and temperature range of 293–373 K.

Preparation of FMO NPs. FMO nanomaterial was synthesized using the Pechini method based on the sol–gel technique. The FMO nanomaterial was synthesized using the Pechini method based on a sol–gel process[1,2]. Many methods are known for the synthesis [3] of FMO nanomaterials: solid-phase precipitation[4], microwave synthesis[5], hydrothermal method [6–10], "green synthesis" method[3,11,12], urea combustion[13], methods based on sol-gel formation[3,14–16], etc. Among these methods, the reason for choosing the Pechini (sol-gel-based) method is; its formation of a homogeneous system with a uniform distribution characteristic of a complex oxide, the stoichiometrically accurate product and the obtaining of a material up to nano-sized. In this method, Mn^{2+} and Fe^{3+} ions form metal citrates with citric acid, which are reacted with ethylene glycol to form a uniform gel-like organic polymer. In this method, duration (6 hours) calcination temperature is used to obtain a truly crystalline nanoproduct from xerogel. Stoichiometric amounts of metal nitrates were used as precursors: 5.09 g of ferric nitrate nonahydrate ($Fe(NO_3)_3 \cdot 9H_2O$) and 2.47 g of manganese nitrate hydrate ($Mn(NO_3)_2 \cdot xH_2O$) were weighed and dissolved in 10 mL of distilled water. To facilitate complexation and gel formation, 2.0 g of citric acid (acting as a chelating agent) and 2.72 mL of ethylene glycol (density: 1.1 g/mL) were added to the solution in a molar ratio of 1:1.5 with respect to the total metal cation content. The mixture was stirred magnetically at 70–90 °C for 10 minutes to initiate polyesterification and ensure homogeneity. The resulting sol was dried at 120 °C for 12 hours to form xerogel. The dried porous product was then ground into a fine powder and subjected to thermal treatment (calcination) at temperatures ranging from 600 to 1200 °C for 6 hours in air. This process resulted in the formation of a single-phase perovskite-like FMO NPs powder.

Preparation of a tablet for electrophysical measurement. Electrophysical properties were measured according to the methods [15]. The research of electrophysical properties (dielectric constant and electrical resistivity) was carried out by measuring the electrical capacitance of the samples on a commercially available LCR-800 instrument (Taiwan) at an operating frequency of

1 kHz in continuous thermostat mode in dry air with each fixed temperature maintenance time. The research of electrophysical properties (dielectric permittivity and electrical resistance) was carried out by measuring the electrical capacity of the samples on a commercially available device LCR-800 (Taiwan) at an operating frequency of 1kHz continuously in dry air in thermostatic mode with holding time at each fixed temperature. Flat-parallel specimens in the form of disks with a diameter of 10 mm and thickness of 2-6 mm with binder additive (1,5 %) were prefabricated. Pressing was carried out under pressure of 20 kg/cm^2. The obtained disks were fired in the laboratory furnace at 400 ºC for 6 hours. Then thorough double-sided grinding was carried out.

The research of electrophysical properties (dielectric permittivity and electrical resistance) was carried out by measuring the electrical capacity of the samples on a commercially available device LCR-800 (Taiwan) at an operating frequency of 1kHz continuously in dry air in thermostatic mode with holding time at each fixed temperature.

Dielectric permittivity was determined from the electrical capacity of the sample at known values of sample thickness and electrode surface area. A Sawyer–Tower scheme was used to obtain the relationship between the electrical induction D and the electric field strength E. Visual observation of D (E of the hysteresis loop) was performed on a C1-83 oscilloscope with a voltage divider consisting of a 6 mOhm and 700 kOhm resistance and a 0.15 μF reference capacitor. The frequency of the oscillator was 300 Hz. In all temperature studies, the samples were placed in an oven and the temperature was measured with a chromel-alumel thermocouple connected to a B2-34 voltmeter with an error of ±0,1 mV. The rate of temperature change ~5 K/min. The value of dielectric permittivity at each temperature was determined by the formula:

$$\varepsilon=\frac{C}{C_0} \qquad (2)$$

where $C_0=\frac{\varepsilon_0 \cdot S}{d}$ - is the capacitance of the capacitor without the investigated substance (air).

The calculation of the forbidden band width (ΔE) of the investigated substance was determined by the formula [17]:

$$\Delta E=\frac{2kT_1T_2}{0.43(T_2\text{-}T_1)}\lg\frac{R_1}{R_2}, \qquad (3)$$

where K is the Boltzmann constant equal to $8,6173303\cdot10^{-5}$ eV·K^{-1} T, R_1 is the resistance at T_1, R_2 is the resistance at T_2.

Electrophysical measurements of FMO in the range 293-483 K and frequencies equal to 1, 5, and 10 kHz were carried out on the LCR-800 setup (table 1).

Table S1. Electrophysical measurement results of FMO obtained in the temperature range of 293–483 K and at frequencies of 1(a), 5(b), and 10(c) kHz.

1. (a) 1 kHz

T, K	C, nF	R, Ом	ε	lgε	lgR
293	0,01203	7140000	69	1,84	6,85
303	0,01428	7650000	82	1,91	6,88
313	0,03368	6122000	194	2,29	6,79

323	0,45471	1528000	2618	3,42	6,18
333	2,6946	551600	15515	4,19	5,74
343	13,98	202400	80492	4,91	5,31
353	35,935	106500	206902	5,32	5,03
363	59,877	71190	344752	5,54	4,85
373	68,989	60940	397216	5,60	4,78
383	23,856	117700	137355	5,14	5,07
393	4,045	377300	23290	4,37	5,58
403	1,3409	803100	7720	3,89	5,90
413	0,99577	1019000	5733	3,76	6,01
423	1,2394	962900	7136	3,85	5,98
433	2,1793	733000	12548	4,10	5,87
443	4,4446	491800	25591	4,41	5,69
453	8,2124	336200	47284	4,67	5,53
463	15,655	223500	90136	4,95	5,35
473	23,25	177800	133866	5,13	5,25
483	42,211	125800	243037	5,39	5,10

2. (b) 5 kHz

**T, K**	**C, nF**	**R, Ом**	**ε**	**lgε**	**lgR**
293	0,00805	917300	46	1,67	5,96
303	0,00857	1138000	49	1,69	6,06
313	0,01232	1660000	71	1,85	6,22
323	0,06505	986100	375	2,57	5,99
333	0,29631	449000	1706	3,23	5,65
343	1,5673	181100	9024	3,96	5,26
353	4,5167	98900	26006	4,42	5,00
363	8,5497	67130	49226	4,69	4,83
373	9,4993	60740	54694	4,74	4,78
383	2,6127	123900	15043	4,18	5,09
393	0,42406	369100	2442	3,39	5,57
403	0,14287	675400	823	2,92	5,83
413	0,09957	806700	573	2,76	5,91
423	0,10951	769200	631	2,80	5,89
433	0,17002	618900	979	2,99	5,79
443	0,33084	435300	1905	3,28	5,64
453	0,61653	304700	3550	3,55	5,48

463	1,2032	205300	6928	3,84	5,31
473	1,7337	166000	9982	4,00	5,22
483	3,0892	118900	17787	4,25	5,08

3. (c) 10 kHz

**T, K**	**C, nF**	**R, Ом**	**ε**	**lgε**	**lgR**
293	0,00751	332400	43	1,64	5,52
303	0,00791	453400	46	1,66	5,66
313	0,01029	781900	59	1,77	5,89
323	0,03464	677600	199	2,30	5,83
333	0,12193	363700	702	2,85	5,56
343	0,56819	164100	3271	3,51	5,22
353	1,6039	92390	9235	3,97	4,97
363	3,0476	64610	17547	4,24	4,81
373	3,2176	61530	18526	4,27	4,79
383	0,75057	134600	4322	3,64	5,13
393	0,13389	364700	771	2,89	5,56
403	0,05339	563900	307	2,49	5,75
413	0,04037	621000	232	2,37	5,79
423	0,04434	590700	255	2,41	5,77
433	0,06427	503000	370	2,57	5,70
443	0,11619	377600	669	2,83	5,58
453	0,21344	273800	1229	3,09	5,44
463	0,4159	188500	2395	3,38	5,28
473	0,59097	153900	3403	3,53	5,19
483	1,0448	112100	6016	3,78	5,05

Conditions of sample preparation and dielectric measurements (powder samples)

Some of the synthesized specimens were sent to Istanbul University for a separate dielectric analysis with dedicated measurement devices, in order to probe the dielectric properties. In the present study, dielectric analysis was performed on samples in powder forms due to the nature of measurement facility and equipments available in host laboratory.

Dielectric measurements were conducted with a Novocontrol Alpha-A broadband dielectric spectrometer, capable of registering complex dielectric permittivity in the broad frequency range. The powder samples were loaded into a dedicated measurement cell for bulk and powder materials with regard to repeatable filling of the measurement volume as well as reliable electrical contact during measurements.

The measurements were made in the frequency interval from 1 Hz to 3 MHz and a temperature domain of 293-393 K (20-120 °C). A Quatro Cryosystem was incorporated into the measurement system to enable temperature control and stabilization of the samples. During the

experiment, the temperature was ramped up in a stepwise manner and dielectric data collection began when thermal stability was achieved at a target temperature.

Figures

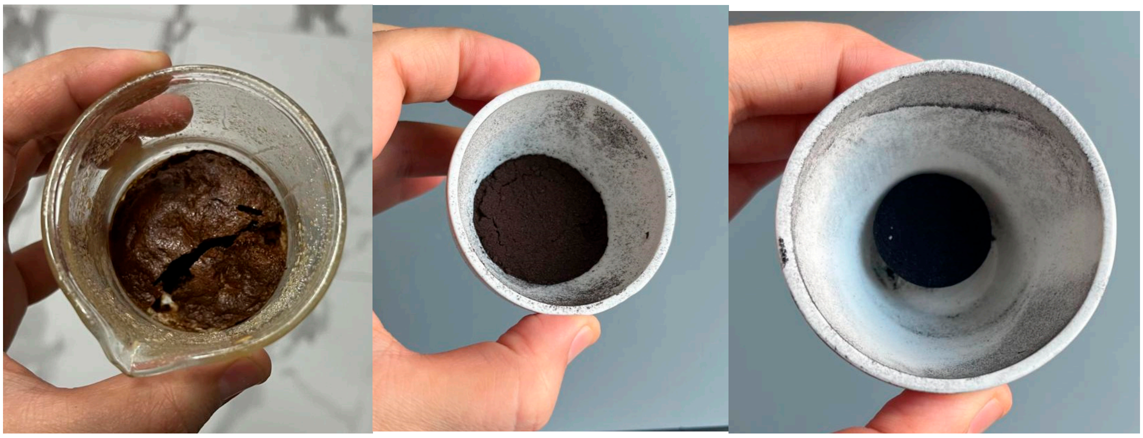

Figure S1. Images of $FeMnO_3$ nanopowder calcined at 120, 600, and 1200 °C

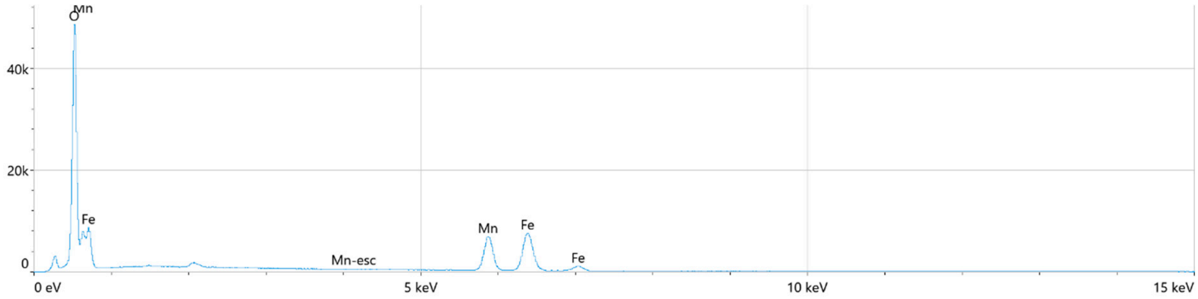

(a)

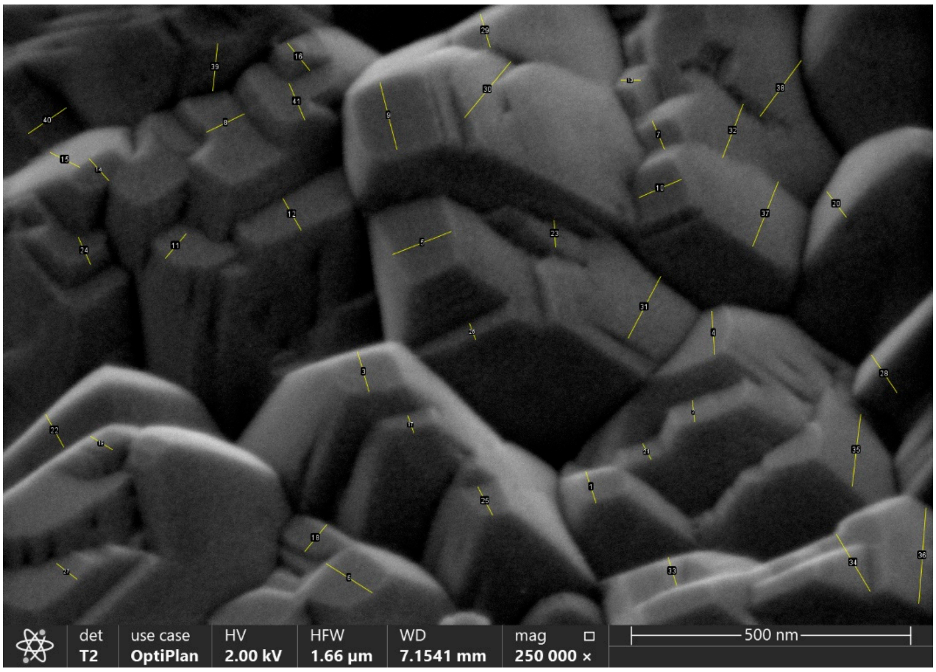

(b)

Figure S2. EDS point analysis results (a) and FESEM images (b) of FMO

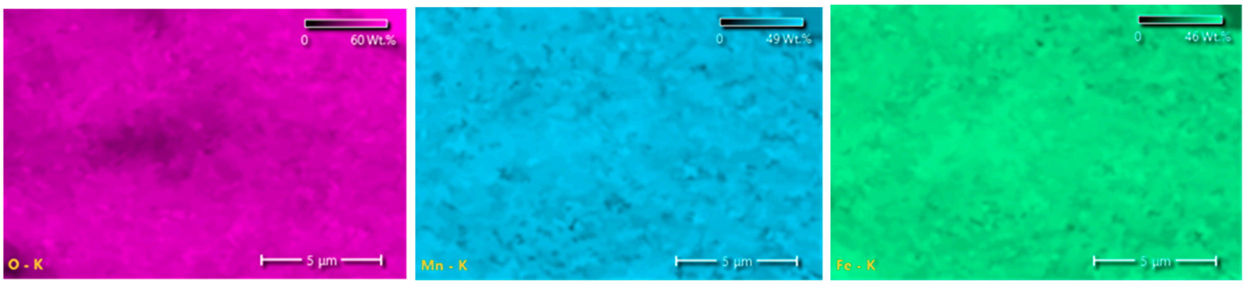

(a)

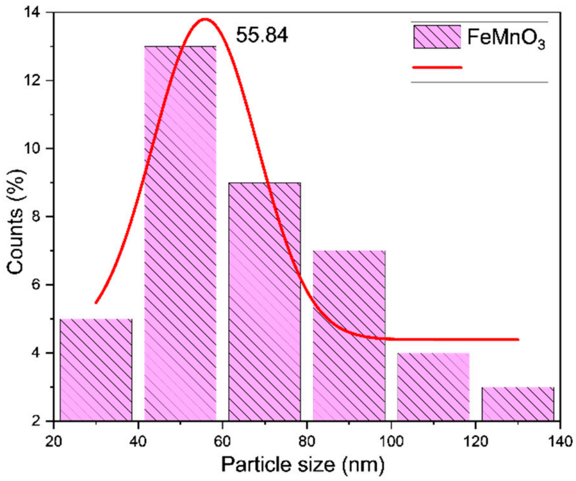

(b)

Figure S3 Mapping analysis results of FMO (a). Histogram of the particle size distribution of the samples determined from FESEM micrographs of FMO (b).

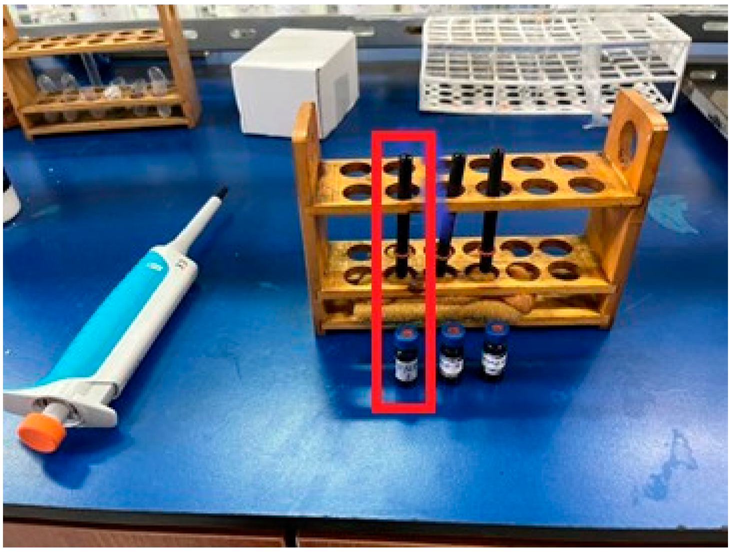

Figure S4. Photo images of the GCE modified with FMO Nps material.

References

1. Mohan, M.; Shetti, N.P.; Aminabhavi, T.M. Perovskites: A new generation electrode materials for storage applications. *J. Power Sources* **2023**, *574*, 233166. https://doi.org/10.1016/j.jpowsour.2023.233166.
2. Ono, L.K.; Juarez-Perez, E.J.; Qi, Y. Progress on Perovskite Materials and Solar Cells with Mixed Cations and Halide Anions. *ACS Appl. Mater. Interfaces* **2017**, *9*, 30197–30246. https://doi.org/10.1021/acsami.7b06001.
3. Abdollah Lachini, S.; Eslami, A.; Chu, Q. Green synthesis of perovskite-type $FeMnO_3$ nanoparticles: Study of its electrochemical hydrogen storage and catalytic activity on the thermal decomposition of ammonium perchlorate. *J. Alloys Compd.* **2025**, *1010*, 177486. https://doi.org/10.1016/j.jallcom.2024.177486.
4. Singh, A.; Verma, R.; Thakur, P.; Thakur, A.; Wan, F. Structural analysis of piezoelectric perovskite materials. In *Biomedical Applications of Perovskites: The Era of Bio-Piezoelectric Systems*; Bentham Science Publishers: Sharjah, United Arab Emirates, 2024; pp. 40–59. https://doi.org/10.2174/9789815256383124010005.
5. Verma, A.S.; Jindal, V.K. ABX_3-type oxides and halides: Their structure and physical properties. In *Perovskites: Structure, Properties and Uses*; Nova Science Publishers: New York, NY, USA, 2010; pp. 463–479.
6. Xu, L.; Li, W.; Luo, J.; Chen, L.; He, K.; Ma, D.; Lv, S.; Xing, D. Carbon-based materials as highly efficient catalysts for the hydrogen evolution reaction in microbial electrolysis cells: Mechanisms, methods, and perspectives. *Chem. Eng. J.* **2023**, *471*, 144670. https://doi.org/10.1016/j.cej.2023.144670.
7. Xie, T.; Lv, Z.; Wang, K.; Xie, G.; He, Y. FeMnO3 nanoparticles promoted electrocatalysts Ni–Fe–P–$FeMnO_3$/NF with superior hydrogen evolution performances. *Renew. Energy* **2020**, *161*, 956–962. https://doi.org/10.1016/j.renene.2020.07.116.
8. Gowreesan, S.; Ruban Kumar, A. Structural, magnetic, and electrical property of nanocrystalline perovskite structure of iron manganite ($FeMnO_3$). *Appl. Phys. A* **2017**, *123*, 689. https://doi.org/10.1007/s00339-017-1302-x.
9. Rayaprol, S.; Kaushik, S.D. Magnetic and magnetocaloric properties of $FeMnO_3$. *Ceram. Int.* **2015**, *41*, 9567–9571. https://doi.org/10.1016/j.ceramint.2015.04.017.
10. Yao, J.; Wu, J.; Yang, Y.; Xiao, S.; Li, Y. Lithium storage performance of coralline-like $FeMnO_3$ anode materials prepared by a facile chemical co-precipitation method. *J. Alloys Compd.* **2020**, *848*, 156444. https://doi.org/10.1016/j.jallcom.2020.156444.
11. Ghosh, D.; Dutta, U.; Haque, A.; Mordvinova, N.E.; Lebedev, O.I.; Pal, K.; Gayen, A.; Mahata, P.; Kundu, A.K.; Seikh, M. Evidence of low temperature spin glass transition in bixbyite type $FeMnO_3$. *Mater. Sci. Eng. B* **2017**, *226*, 206–210. https://doi.org/10.1016/j.mseb.2017.09.022.

12. Nikolic, M.V.; Krstic, J.B.; Labus, N.J.; Lukovic, M.D.; Dojcinovic, M.P.; Radovanovic, M.; Tadic, N.B. Structural, morphological and textural properties of iron manganite ($FeMnO_3$) thick films applied for humidity sensing. *Mater. Sci. Eng. B* **2020**, *257*, 114547. https://doi.org/10.1016/j.mseb.2020.114547.
13. Barros, L.N.L.C.; Araujo, R.N.D.; Nascimento, E.P.D.; Gama, A.J.D.A.; Neves, G.A.; Torres, M.A.M.; Menezes, R.R. Influence of Fast Drying on the Morphology of α-Fe_2O_3 and $FeMnO_3$/α-Fe_2O_3 Fibers Produced by Solution Blow Spinning. *Nanomaterials* **2024**, *14*, 304. https://doi.org/10.3390/nano14030304.
14. Vinoth, C.; Gajendiran, J. Structural, Electrical, and electrochemical performance of $FeMnO_3$ nanostructures for electrochemical storage devices and battery applications. *Inorg. Chem. Commun.* **2025**, *172*, 113746. https://doi.org/10.1016/j.inoche.2024.113746.
15. Mantilla, J.C.; Nagamine, L.C.C.M.; Cornejo, D.R.; Cohen, R.; De Oliveira, W.; Souza, P.E.N.; Silva, S.W.D.; Aragón, F.F.H.; Gastelois, P.L.; Morais, P.C.; et al. Structural, morphological, and magnetic characterizations of $(Fe_{0.25}Mn_{0.75})_2O_3$ nanocrystals: A comprehensive stoichiometric determination. *Mater. Chem. Phys.* **2024**, *328*, 129943. https://doi.org/10.1016/j.matchemphys.2024.129943.
16. Vinoth, C.; Ramana Ramya, J.; Gajendiran, J.; Gnanam, S.; Gokul Raj, S.; Ramesh Kumar, G.; Karthikeyan, M. Structural, magnetic, antimicrobial and hemolysis properties of sol–gel derived iron manganese tri oxide ($FeMnO_3$) nanostructures. *Inorg. Chem. Commun.* **2023**, *154*, 110952. https://doi.org/10.1016/j.inoche.2023.110952.
17. Azim, F.; Mohapatra, J.; Joshi, P.; Liu, J.P.; Mishra, S.R. Study of magnetic and magnetocaloric properties of $FeMnO_3$ compound. *MRS Adv.* **2025**, *10*, 1196–1202. https://doi.org/10.1557/s43580-025-01142-w.
